# Timing and intensity of heat and drought stress determine wheat yield losses in Germany

**DOI:** 10.1371/journal.pone.0288202

**Published:** 2023-07-25

**Authors:** Ludwig Riedesel, Markus Möller, Peter Horney, Burkhard Golla, Hans-Peter Piepho, Timo Kautz, Til Feike

**Affiliations:** 1 Julius Kühn Institute (JKI) – Federal Research Centre for Cultivated Plants, Institute for Strategies and Technology Assessment, Kleinmachnow, Germany; 2 Julius Kühn Institute (JKI) – Federal Research Centre for Cultivated Plants, Institute for Crop and Soil Science, Braunschweig, Germany; 3 Institute of Crop Science, Biostatistics Unit, University of Hohenheim, Stuttgart, Germany; 4 Humboldt University of Berlin, Thaer-Institute of Agricultural and Horticultural Sciences, Berlin, Germany; Arab Academy for Science Technology and Maritime Transport, EGYPT

## Abstract

Crop yields are increasingly affected by climate change-induced weather extremes in Germany. However, there is still little knowledge of the specific crop-climate relations and respective heat and drought stress-induced yield losses. Therefore, we configure weather indices (WIs) that differ in the timing and intensity of heat and drought stress in wheat (*Triticum aestivum L.*). We construct these WIs using gridded weather and phenology time series data from 1995 to 2019 and aggregate them with Germany-wide municipality level on-farm wheat yield data. We statistically analyze the WI’s explanatory power and region-specific effect size for wheat yield using linear mixed models. We found the highest explanatory power during the stem elongation and booting phase under moderate drought stress and during the reproductive phase under moderate heat stress. Furthermore, we observed the highest average yield losses due to moderate and extreme heat stress during the reproductive phase. The highest heat and drought stress-induced yield losses were observed in Brandenburg, Saxony-Anhalt, and northern Bavaria, while similar heat and drought stresses cause much lower yield losses in other regions of Germany.

## Introduction

Wheat (*Triticum aestivum L.*) stands out for its high-yielding varieties, high level of disease resistance, nutritional properties, and excellent baking characteristics [1]. Consequently, wheat covers the largest production area globally [2] and is one of the most important crops for global food security [3]. Due to a growing world population, economic growth, and changing dietary habits, the global demand for wheat is continuously increasing [4, 5]. As the second largest wheat producer in the European Union with high yield levels in international comparison [2], Germany plays an important role in the global wheat supply. However, while wheat yields have continuously increased in Germany in recent decades [6], stagnation of yields has been observed in Western Europe, including Germany, in recent years [7]. This stagnation can be explained by several factors, such as a slight extensification of production [8], reduced breeding progress [9], the expansion of winter wheat cultivation to more marginal sites [10] and, in particular, adverse effects of global climate change [11].

Against this background, wheat yield losses due to weather extremes are increasing in Germany [12–15], with heat stress (high-temperature stress) and drought stress (soil water stress) considered the most important abiotic stress factors [15–23]. However, the relationships between regional weather extremes and on-farm yield losses are still insufficiently described for wheat in Germany [22, 24–26]. Hence, a better understanding of the spatiotemporal effects of heat and drought stress on wheat yields is required [13, 25].

Weather Indices (WIs) can help explain the impact of extreme weather events on crop yields [13, 27–30]. WIs are based on the calculation of statistical indicators (e.g., temperature sum) or the number of thresholds exceeded by an indicator (e.g., days with a maximum temperature above 31°C) within a reference period relevant for crop growth. A particular challenge in defining relevant WIs is the complexity and limited knowledge of the relationships between crop yields, regional site conditions, and changing weather conditions during sensitive growing periods [13, 26, 31]. In this regard, high quality spatiotemporal data are essential for designing suitable WIs [29]. This challenge is particularly evident in relation to 1. the spatial accuracy of yield data sets, 2. the consideration of physiologically relevant and spatiotemporally accurate crop growing periods and 3. the selection of suitable threshold values:

1. Some studies use yield data on a national level but at low spatial resolution [13, 23, 27, 28], and others examine yield data as point data for individual locations [29, 31, 32] or smaller regions [15, 16], but only very few studies use high-resolution on-farm yield data collected nationwide [14, 33].
2. Most studies assume fixed growing periods based on calendric days [13], ignoring regional and temporal differences in plant development. Others follow more dynamic approaches by calculating phenological phases using growing degree day (GDD) methods [31–35]. Deriving GDD values allows a more spatiotemporally dynamic approach, but it is limited to assumptions such as sowing date [36]. Time series of gridded phenological observation data sets create the possibility of computing WIs within phenological phases for each year and location, promising to better describe the crop-climate relations than static calendric approaches [29, 36]. Thus far, however, there have been hardly any studies that make use of such data sets [14, 16].
3. Determining suitable yield-effective threshold values is challenging and handled very differently in the literature [23, 25, 27, 37]. Some studies define their thresholds according to plant physiological reactions based on controlled experiments [38] ignoring possible spatial and temporal differences in their occurrence out in the field [31, 32, 39]. Other studies, do not take plant physiology into account and base the threshold values on the extremes of local weather phenomena [14, 23, 27, 40]. To the best of our knowledge, no study has compared the spatial effects of moderate vs. extreme stress nationwide.

With this in mind, the aim of this study is to analyze wheat yield effects with WIs that differ in the intensity and timing of heat and drought stress based on region-specific interpolated weather and phenology data. Therefore, our objectives are as follows:

1. To analyze the differences in explanatory power between the timing and intensity of heat and drought WIs
2. To evaluate the regional differences in heat and drought stress-related yield effects on winter wheat

Consequently, we create WIs that differ in the intensity and timing of heat and drought stress based on region-specific interpolated weather and phenology data. We then evaluate these WIs for their explanatory power and region-specific effect size using mixed linear models. Based on this, we calculate and present the region-specific yield effects of the different WIs.

## Material and methods

### Study design

**Fig 1:**
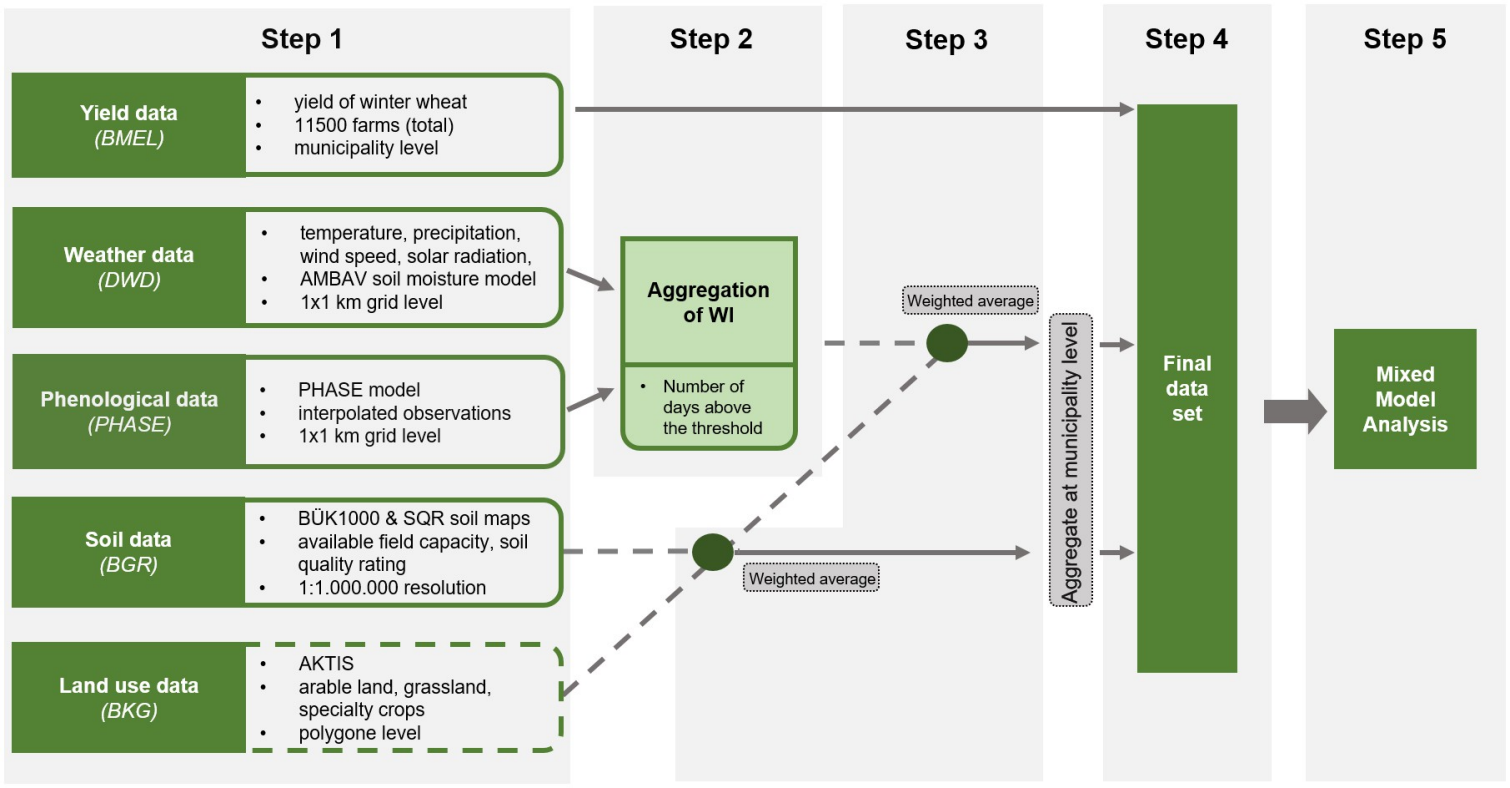
Overview of the study design and respective five working steps: From 1) listing the data sets used, 2) aggregation of spatiotemporal weather indices (WIs), 3) spatial aggregation of the soil and WI data, 4) integration of all data into one comprehensive data set and 5) statistical analysis.

Fig 1 illustrates the study design along with the various data integration steps:

1. We compile all required data sets, including yield, weather, phenology, soil, and land use data sets (Sec. Data sets).
2. We blend phenological data with weather data and derive a set of dynamic WIs based on the resulting nationwide 1 × 1 km^2^ grid data base (Sec. Dynamic WI configuration).
3. We merge the soil data and WI data with the land use data for each grid cell. That way, we consider only the weather and soil data for cropland in each grid cell, excluding other land use types (e.g., grassland, forests, and specialty crops) from the analysis. We then aggregate the data at the municipality level, using weighted averages depending on the share of cropland per grid cell. Since the municipality boundaries have been changed in several municipal reforms, with respect to year, we base the WI aggregation on the geometries of the respective last reform.
4. We assign municipality-specific WIs and soil parameters from Step 3 to each farm-specific yield data point for each year and municipality.
5. Finally, we statistically analyze the resulting data using a mixed model approach (Sec. Statistical analysis).

### Data sets

#### Yield data

The Federal Ministry of Food and Agriculture (BMEL) provided data from the Farm Accountancy Data Network (FADN) for this study [41]. In the FADN, the crop-specific yield data (in dt ha^−1^) and cropland size (in ha) of approximately 11,500 representative farms were collected annually between 1995 and 2019 and analyzed anonymously. Each farm is assigned a municipality index and a farm code so that it is possible to distinguish the farms from each other at the municipality level without sharing their identity and exact location.

#### Weather data

The German Weather Service (DWD) provided daily meteorological data, including minimum, mean and maximum temperature, cumulative precipitation, mean wind speed, and solar radiation. Daily measures from weather stations have been available since 1993 and interpolated to a 1 × 1 km^2^ grid-based resolution [42]. Furthermore, the DWD also provided drought-related data since 1993 on the plant available water capacity (*PAWC*) in the 0 to 60 cm soil layer generated by the AMBAV model [43]. The model inputs include plant-specific height, the leaf area index, rooting depth and density, and water fluxes in the soil.

#### Phenology data

The DWD operates a phenological network of annual and immediate observers. Approximately 1200 observers monitor 160 phenological phases of wild and cultivated plants. The observations are mapped according to a standardized protocol [44]. The PHASE model was developed to interpolate the phenological observations for the entire territory of Germany [45]. Since temperature can be considered the most crucial factor influencing phenology in Central Europe [46], the model combines the concept of GDDs with a geostatistical interpolation procedure. An essential application of the data sets is the derivation of dynamic time windows for specific years, phases, and test sites [47], which can be defined as growing periods between two crop-specific, consecutively observed phenological events [48]. As shown by Bucheli et al. [16], considering adverse weather conditions during specific phenological phases vs. static calendric time windows helps explain weather-yield relations.

#### Soil quality data

We use the Soil Quality Rating (SQR) soil map from the BGR to describe soil quality. The SQR classifies soils globally according to their suitability for agricultural land use and yield potential. This classification is available at a 250 × 250 m^2^ grid-based resolution [49].

#### Land use data

The ATKIS data set from the Federal Agency for Cartography and Geodesy (BKG) enables GIS-based area surveys. Hence, we can carry out area-wide quantification of land use types (i.e., cropland, grassland, and specialty cropland) at the polygon level [50].

### Dynamic WI configuration

To configure a WI, we first choose the crop (i.e., winter wheat) and weather event (i.e., heat and drought stress), set the regional expanse (i.e., nationwide), and depict the spatial aggregation (i.e., municipality level). Second, we express the configuration conditions in setting the parameter (i.e., daily maximum temperature, daily average plant available water capacity) and the index (i.e., accumulated days above the threshold). Finally, we define timing and intensity to build up the WI structure.

**Fig 2:**
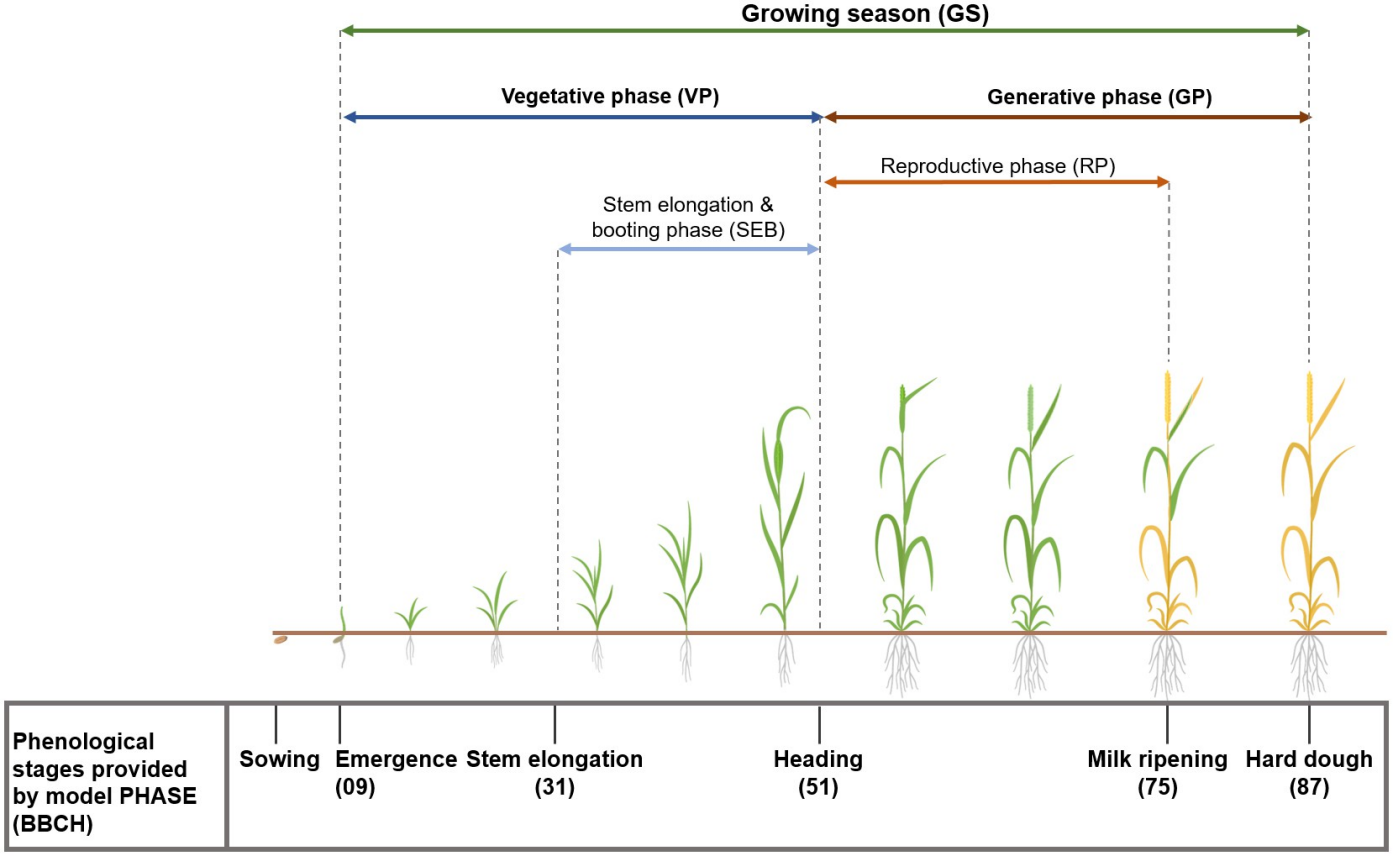
Phenological phases with values according to the BBCH scale available from the PHASE model and growing phases (colored) for winter wheat along the vegetation period.

Timing refers to the year- and location-specific phenological development phases [15, 31, 51]. We select three growing periods where heat and drought stress are of specific crop physiological relevance [19, 20, 52]. Hence, we select the “stem elongation and booting phase” (SEB; BBCH 31—50), “reproductive phase” (RP; BBCH 51—75), and “generative phase” (GP; BBCH 51—87). We illustrate the respective growing phases in Fig 2.

Intensity refers in our study to a fixed threshold value of a weather variable that must be exceeded for the WIs to take effect [36]. We base the thresholds on crop physiological understanding, describing moderate, severe, and extreme stress intensities. We calculate the heat-related WIs from the accumulated number of days with a maximum temperature exceeding 27, 29 or 31°C, i.e., *T*_*max*_ > [27, 29, 31]°C [20, 31, 32]. We further calculate the drought-related WIs from the accumulated number of days with plant available water (PAW) below 50, 30 and 10% of the plant available water capacity (PAWC), i.e., *PAWC* < [10, 30, 50]% [32, 54]. We form all *timing* × *intensity* combinations and thus obtain 18 WIs with moderate, severe, and extreme intensities for the three growing periods (Table 1).

**Table 1:** List of moderate, severe, and extreme heat and drought WIs for the growing periods stem elongation and booting phase (SEB), reproductive phase (RP) and generative phase (GP).

Name	Weather event	Intensity	Intensity threshold	Timing
H27_SEB	heat	moderate	*T*_*max*_ > 27°C	BBCH 31–50
H29_SEB	heat	severe	*T*_*max*_ > 29°C	BBCH 31–50
H31_SEB	heat	extreme	*T*_*max*_ > 31°C	BBCH 31–50
H27_RP	heat	moderate	*T*_*max*_ > 27°C	BBCH 51–75
H29_RP	heat	severe	*T*_*max*_ > 29°C	BBCH 51–75
H31_RP	heat	extreme	*T*_*max*_ > 31°C	BBCH 51–75
H27_GP	heat	moderate	*T*_*max*_ > 27°C	BBCH 51–87
H29_GP	heat	severe	*T*_*max*_ > 29°C	BBCH 51–87
H31_GP	heat	extreme	*T*_*max*_ > 31°C	BBCH 51–87
D50_SEB	drought	moderate	<50% PAWC	BBCH 31–50
D30_SEB	drought	severe	<30% PAWC	BBCH 31–50
D10_SEB	drought	extreme	<10% PAWC	BBCH 31–50
D50_RP	drought	moderate	<50% PAWC	BBCH 51–75
D30_RP	drought	severe	<30% PAWC	BBCH 51–75
D10_RP	drought	extreme	<10% PAWC	BBCH 51–75
D50_GP	drought	moderate	<50% PAWC	BBCH 51–87
D30_GP	drought	severe	<30% PAWC	BBCH 51–87
D10_GP	drought	extreme	<10% PAWC	BBCH 51–87

### Spatial aggregation

**Fig 3:**
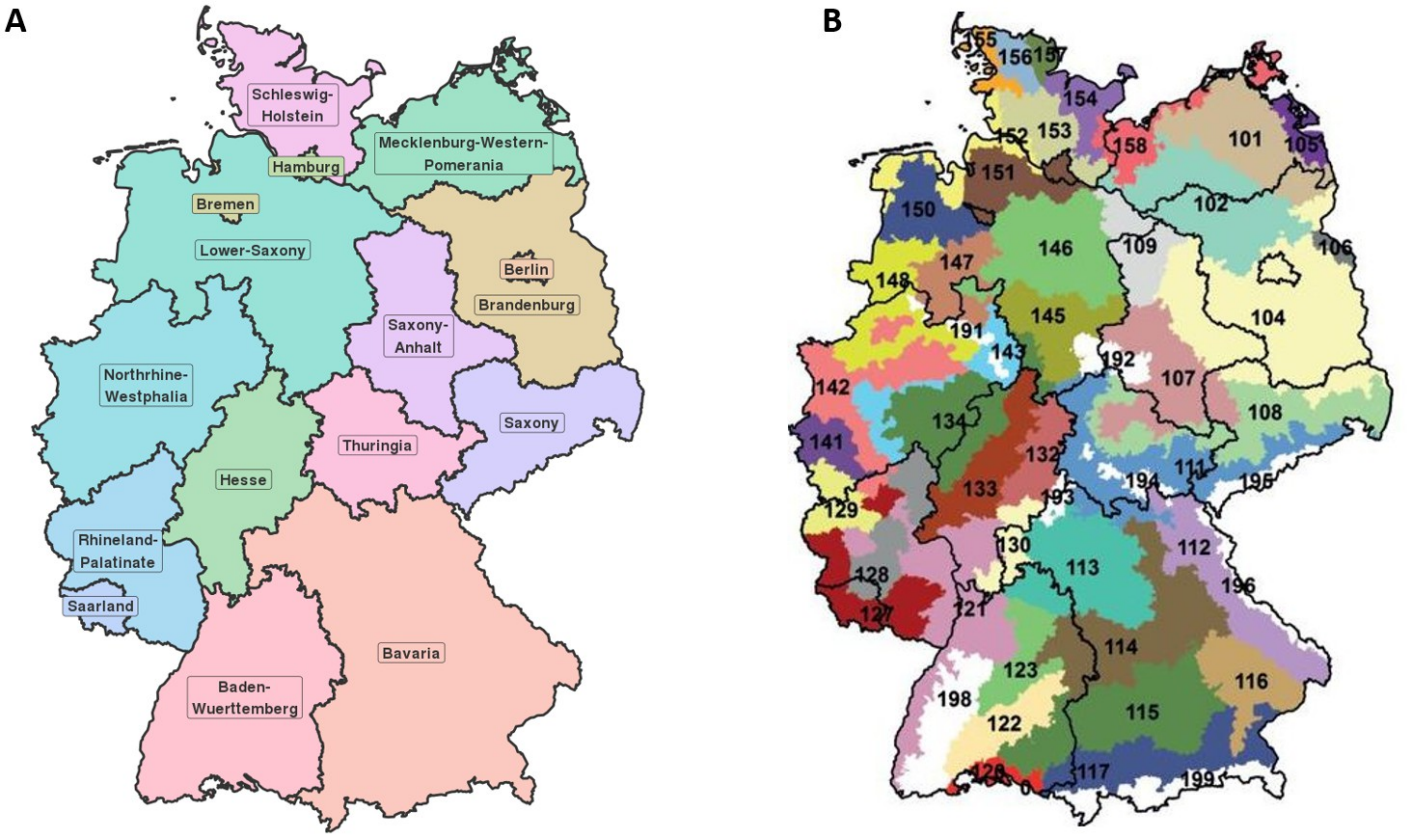
**A** Federal states in Germany. **B** Fifty Soil Climate Regions (SCRs) within the federal states of Germany. The designation of the numbered SCRs is shown in Table A in S1 File. The maps were reprinted from [53] under a CC BY license, with permission from [GeoBasis-DE/ BKG], original copyright [2023].

To evaluate the weather-yield relations while considering spatial differences, we utilize the 50 SCRs within Germany in our analysis according to Roßberg et al. [55]. SCRs represent regions of similar agricultural growth conditions. SCRs were derived considering soil (i.e., the weighted soil quality) and weather information (i.e., mean monthly temperature and mean monthly precipitation sum for the period March–August in 1981–2000) at the municipality level. We present the federal states and SCR within the federal states in Fig 3. Furthermore, we list a detailed description of the individual SCRs in Table A in S1 File.

### Statistical analysis

#### Mixed linear model

We use linear mixed models based on Bönecke et al. and Hadasch et al. [31, 32]. In the first part of the model, we depict the fixed regression terms (first line) and the random main and interaction effects (second line). Our model can be expressed according to Eq (1)

$$\begin{array}{ccc} z_{jklm} & =\mu +\gamma t_{j}+\varsigma n_{l}+S_{m}+\delta {\left(wS\right)}_{jlm} & \\ & +M_{l}+Y_{j}+F_{kl}+{\left(MY\right)}_{lj}+{\left(MF\right)}_{lk}+{\left(YF\right)}_{jk}+e_{jklm} \end{array}$$ (1)

where *z*_*jklm*_ represents the mean yield of the *j*-th harvesting year in the *k*-th farm location in the *l*-th municipality of the *m*-th SCRs. *μ* is the overall intercept, *γt*_*j*_ represents the genetic and nongenetic time trend where *γ* is a fixed regression coefficient for the time trend and *tj* is the continuous covariate for the *j*-th harvesting year. The covariate *ς*_*nl*_ accounts for the continuous soil quality ratings (SQRs) from 1 to 100 points, where *ς* is the regression coefficient, and *n*_*l*_ represents the SQR value for the *l*-th municipality. *S*_*m*_ is a categorical covariate with 50 levels (m) considering the different SCRs. *δ*(*wS*)_*jlm*_ is the interaction term between the WI and SCR, where *δ* is the fixed regression coefficient for the interaction of *w*_*jl*_ and *S*_*m*_, with *w*_*jl*_ representing the continuous covariate of the WI in the j-th year and l-th municipality. For the random effects, *M*_*l*_ is the main effect of the *l*-th municipality, *Y*_*j*_ is the main effect of the j-th year, and *F*_*kl*_ is the main effect of the *k*-th farm within the *l*-th municipality. (*MY*)_*lj*_ is the *lj*-th municipality × year interaction effect, (*MF*)_*lk*_ is the *lk*-th municipality × farm interaction effect, (*YF*)_*jk*_ is the *jk*-th year × farm interaction effect and *e*_*jklm*_ is a random residual.

#### Explanatory power

To quantify the explanatory power of the various heat and drought WIs, we analyze the variance reduction (VR) of each WI by estimating the coefficient of determination (*R*^2^) for mixed models following Piepho [56]. In this regard, we analyze the variance of the random effects (*M*, Eq (1)) twice—first without (*Var*_*y*(*M*_−*x*_)_) and second with (*Var*_*y*(*M*_+*x*_)_) the WI-term *δ*(*wS*)_*jlm*_ as our variable under assessment. Next, we derive the VR (%*Var*_*y*_) of every WI one by one by calculating the relative change () in the total variance of the random effects between the two models as described in Eq (2).

$$\begin{array}{c} \%Var_{y}=\frac{Var_{y(M_{-x})}-Var_{y(M_{+x})}}{Var_{y(M_{-x})}}\times 100 \end{array}$$ (2)

#### Region-specific effect size and yield effects

We analyze the region-specific effect size of each WI in each SCR individually by deriving the estimated coefficients (*δ*) of the regression term *δ*(*wS*)_*jlm*_. To obtain the resulting annual yield effects on municipality level, we multiply the estimated coefficient (*δ*) per SCR (*S*_*m*_) with the respective WI value (*w*_*jl*_).

#### Selection of covariates

We again use the VR with the coefficient of determination for mixed models after Piepho [56] to select covariates (Sec. Explanatory power). In this context, we select a covariate when the VR of *M* + 1 is at least -0.5% compared to the baseline model given in Eq (3). below:

$$\begin{array}{ccc} z_{jkl} & =\mu & \\ & +M_{l}+Y_{j}+F_{kl}+{\left(MY\right)}_{lj}+{\left(MF\right)}_{lk}+{\left(YF\right)}_{jk}+e_{jkl} \end{array}$$ (3)

We also compare the models according to the Akaike information criterion (AIC) and select the covariates if, in addition to a *VR* > 0.5%, they have a smaller AIC value than the baseline model [57]. To guarantee comparability with the AIC methodology, we follow Faraway [58] and change the estimation scheme of the mixed linear models from restricted maximum likelihood (REML) to maximum likelihood (ML). In addition, we also test the variance inflation factor (VIF) to check for multicollinearity. The VIF measures how much the variance of a regression coefficient is increased due to collinearity. If the VIF exceeds five, the multicollinearity is considerably high, and we reject the covariate [59]. To apply the VIF, we need to remove the random effects from the models. We list the covariates used in the analyses and their VR and VIF values in Table C in S1 File.

## Results

### Explanatory power of heat and drought WIs

**Fig 4:**
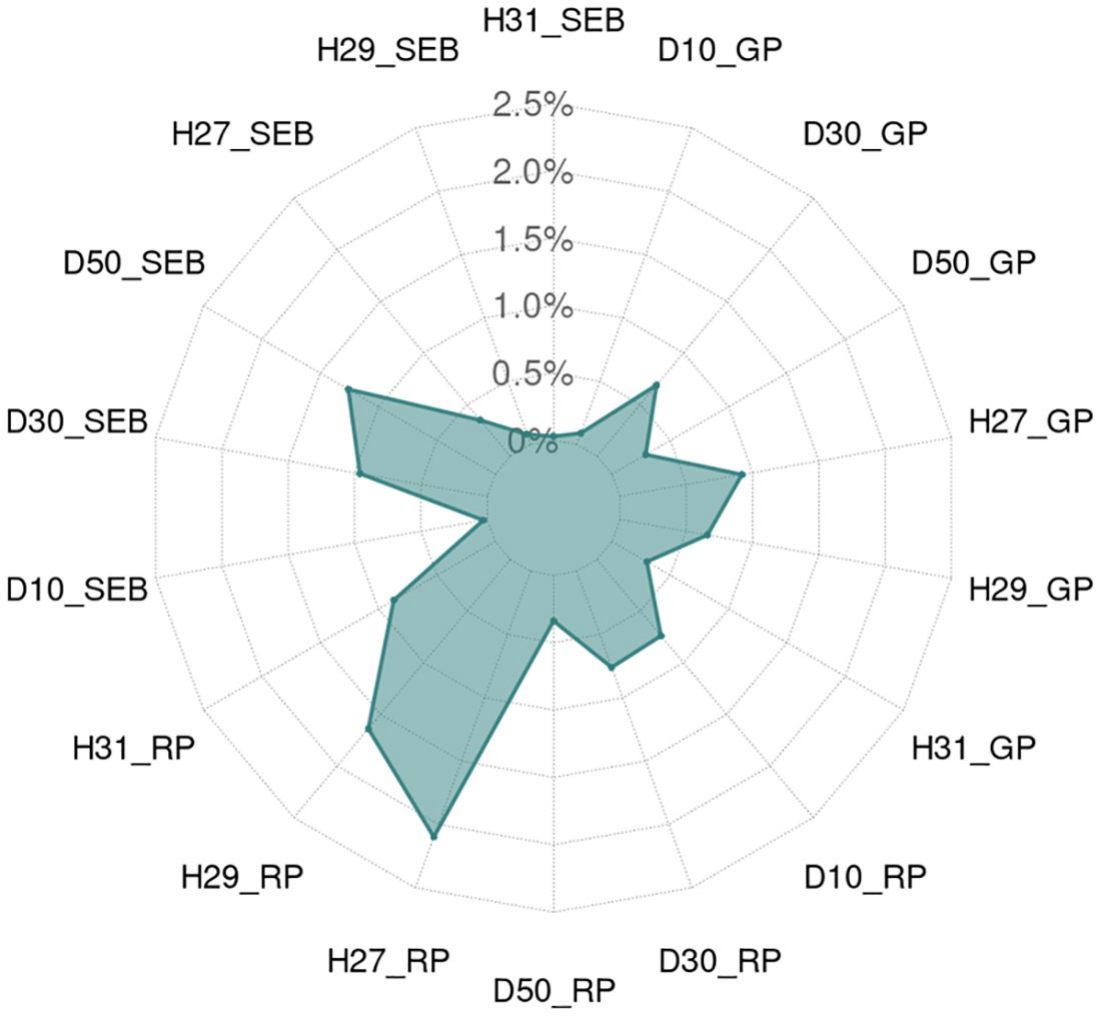
Variance reduction (VR) of the analyzed heat and drought WIs. For WI abbreviations, see Table 1. VR is calculated based on (Eq (2)).

In Fig 4, we observe the highest VR for the moderate heat WI during the reproductive phase with a *T*_*max*_ above 27°C (H27_RP, -2.10%), followed by the severe heat WI with a *T*_*max*_ above 29°C (H29_RP, -1.64%), and the extreme heat WI with a *T*_*max*_ above 31°C (H31_RP, -0.87%). Looking at the heat WIs during the generative phase, the moderate heat WI with a *T*_*max*_ above 27°C again shows the highest VR (H27_GP, -0.92%), followed by the severe heat WI (H29_GP, -0.65%) and the extreme heat WI (H31_GP, -0.30%). During the stem elongation and booting phase, all heat WIs show comparatively low VR values, where the moderate WI with a *T*_*max*_ above 27°C (H27_SEB) ranks highest (-0.35%), followed by the severe WI with a *T*_*max*_ above 29°C (H29_SEB, 0.08%) and the extreme WI with a *T*_*max*_ above 31°C (H31_SEB, 0.02%).

In contrast to the pattern observed for heat WIs, the highest VR is observed for drought during the stem elongation and booting phase, where the moderate drought WI with PAW below 50% *PAWC* (-1.26%; D50_SEB) ranks highest, followed by the severe drought WI with PAW below 30% *PAWC* (-0.96%, D30_SEB). In comparison, the extreme drought WI with PAW below 10% *PAWC* shows an almost zero VR (-0.03%; D10_SEB). During the reproductive phase, the severe and extreme drought WIs at PAW below 30% *PAWC* (D30_GP) and PAW below 10% *PAWC* show approximately double the VR (-0.76%, -0,74%) of the moderate drought WI at PAW below 50% *PAWC* (D50_RP, -0.34%). For drought stress during the generative phase, the severe drought WI has the highest VR at PAW below 30% *PAWC* (D30_GP, -0.69%), followed by the moderate drought WI at PAW below 50% *PAWC* (D50_GP,—0.29%), while the extreme drought WI at PAW below 10% *PAWC* shows almost no VR (D10_GP, -0.09%). We list the VR of every WI in Table B in S1 File.

### Region-specific effect size and yield effects of heat and drought WIs

Below, we analyze the region-specific effect size and yield effect of the drought WIs during the stem elongation and booting phase and the heat and drought WIs during the reproductive phase. As the heat WIs during the stem elongation and booting phase and the heat and drought WIs during the generative phase show almost no explanatory power across all intensities, we do not further illustrate their regional yield effects. However, we list the model outputs for all WIs in Tables D-V in S1 File. All model outputs are based on (1).

#### Drought effects during the stem elongation and booting phase (BBCH 31–50)

**Fig 5:**
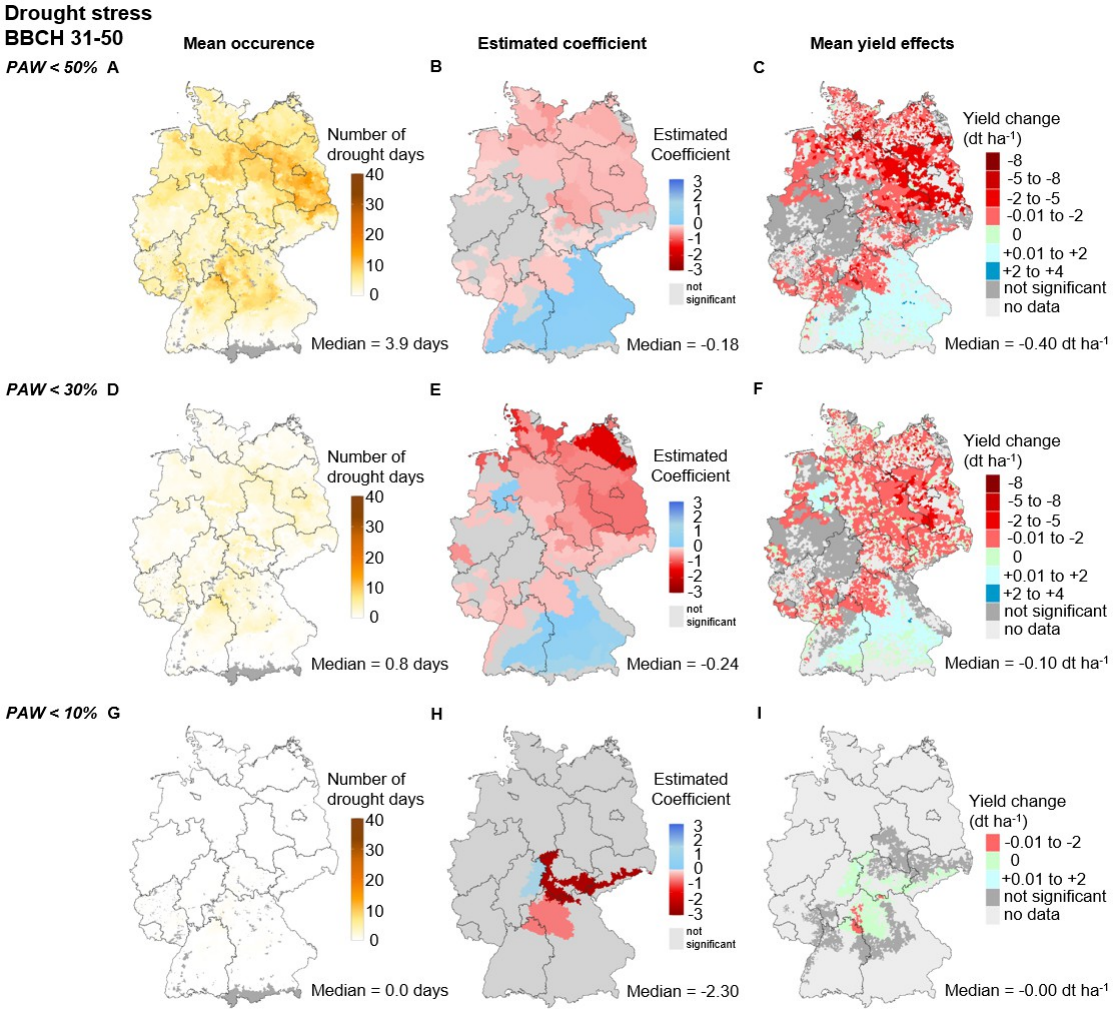
Drought stress during the stem elongation and booting phase (SEB, BBCH 31–50) for moderate (<50% *PAWC*), severe (<30% *PAWC*) and extreme (<10% *PAWC*) stress intensities. **Mean occurrence (A, D, G)** describes the average number of days above the respective thresholds between 1995 and 2019 at the municipality level. **Estimated coefficients (B, E, H)** describe the WI x SCR regression coefficients of Eq 1 for each SCR. Nonsignificant values are given in dark gray. Significant values are given in red (negative effect) or blue (positive effect). The regression coefficients and p-values are displayed in Tables P-R in S1 File. **Mean yield effects (C, F, I)** describe the average yield change in dt ha^−1^ per municipality between 1995 and 2019. The median values below each map refer to the median of all municipalities’ SCRs with significant effects on the respective index. The maps were reprinted from [53] under a CC BY license, with permission from [GeoBasis-DE/ BKG], original copyright [2023].

The median number of drought days during the stem elongation and booting phase ranges from 3.9 days at PAW below 50% *PAWC* (i.e., moderate WI) to 0.8 days at PAW below 30% *PAWC* (i.e., severe WI), down to 0 days at PAW below 10% *PAWC* (i.e., extreme WI). Moreover, the number of drought days is highest in Brandenburg and the northern regions of Lower Saxony and Saxony-Anhalt, as well as Bavaria and Baden-Wuerttemberg. At the same time, it is the lowest in the southern regions of Bavaria and Baden-Wuerttemberg (Fig 5A, 5C and 5G).

The Germany-wide median effect size increases with increasing stress intensity and ranges from -0.18 dt ha^−1^ day above threshold^−1^ (<50% *PAWC*), to -0.24 dt ha^−1^ day above threshold^−1^ (<30% *PAWC*), down to -2.31 dt ha^−1^ day above threshold^−1^ (<10% *PAWC*). The moderate and severe WIs both show a north-south gradient in the effect size, with estimated coefficients being significantly negative in the north and east and positive in the south. In the west, both WIs show no significant effects in many regions (Fig 5B and 5E). The extreme WI reveals only three regions in the center of Germany with significant effects and estimated coefficients ranging up to -9.3 dt ha^−1^ day above threshold^−1^.

Drought in the stem elongation and booting phase exhibits the most substantial yield losses under moderate drought intensities. For the moderate WIs, we observe the highest adverse yield effects (2—5 dt ha^−1^) in the north and southeast of Saxony-Anhalt and the predominant municipalities of Brandenburg. The remaining regions with significantly adverse yield effects show average yield losses between 0.01 and 2 dt ha^−1^. In Bavaria and Baden-Wuerttemberg, we note yield gains between 0.01 and 2 dt ha^−1^ (Fig 3C). An increase in intensity from moderate to severe drought thresholds causes a considerable reduction of the observed number of days above the threshold (−80%) but a minor increase in the effect strength (+33%). Thus, the captured yield effects are comparably low for severe drought intensities, where we observe the highest yield losses (2—5 dt ha^−1^) in a few municipalities in southwestern Brandenburg and southeastern and northeastern Saxony-Anhalt. The remaining regions with significant negative effect strengths reveal yield losses between 0.01—2 dt ha^−1^. In central Bavaria and eastern Baden-Wuerttemberg, yield increases between 0.01—2 dt ha^−1^ are still prevalent (Fig 5F). As the number of days above the threshold is zero for extreme drought intensities, yield effects are also zero for extreme drought during the stem elongation and booting phase.

#### Drought effects during the reproductive phase (BBCH 51–75)

**Fig 6:**
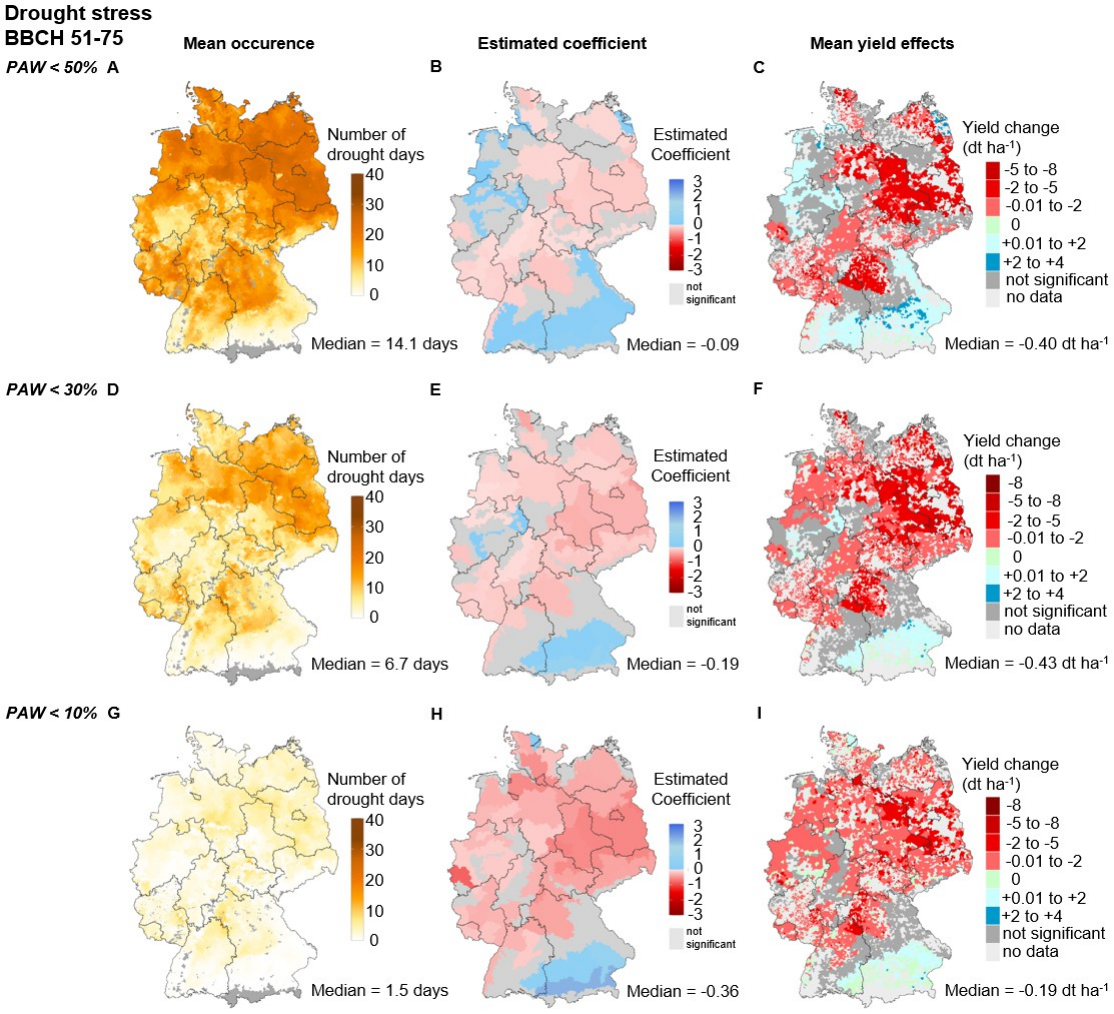
Drought stress during the reproductive phase (RP, BBCH 51–75) for moderate (<50% *PAWC*), severe (<30% *PAWC*) and extreme (<10% *PAWC*) intensities. **Mean occurrence (A, D, G)** describes the average number of days above the respective thresholds between 1995 and 2019 at the municipality level. **Estimated coefficients (B, E, H)** describe the WI x SCR regression coefficients of Eq 1 for each SCR. Nonsignificant values are given in dark gray. Significant values are given in red (negative effect) or blue (positive effect). The regression coefficients and p values are displayed in Tables M-O in S1 File. **Mean yield effects (C, F, I)** describe the average yield change in dt ha^−1^ per municipality between 1995 and 2019. The median values below each map refer to the median of all municipalities’ SCRs with significant effects on the respective index. The maps were reprinted from [53] under a CC BY license, with permission from [GeoBasis-DE/ BKG], original copyright [2023].

The average number of drought days during the reproductive phase ranges from 0 to 39. The Germany-wide median decreases with increasing drought intensities from 14.1 days at PAW below 50% *PAWC* (i.e., moderate WI) to 6.7 days at PAW below 30% *PAWC* (i.e., severe WI), and down to 1.5 days at PAW below 10% *PAWC* (i.e., extreme WI). Most drought days occur in northeastern Germany and the northern regions of Bavaria and Baden-Wuerttemberg, and the fewest drought days occur in southern Bavaria and Baden-Wuerttemberg (Fig 6A, 6C, 6G).

The median effect size increases with increasing intensity (i.e., decreasing *PAWC*) from −0.09 dt ha^−1^ day above threshold^−1^ at PAW below 50% *PAWC* to −0.19 dt ha^−1^ day above threshold^−1^ at PAW below 30% *PAWC* and up to −0.36 dt ha^−1^ day above threshold^−1^ at PAW below 10% *PAWC*. All drought WIs show a similar pattern of region-specific effect size, where significant adverse yield effects are predominantly seen in the north, east, and southwest (Fig 6A and 6C). The number of regions with significant adverse yield effects increases in the west and south with increasing intensity. The moderate WI reveals regions with positive effect size in the south and the northwest. Positive effect sizes with PAW below 10% *PAWC* occur only in southern Bavaria (Fig 6H).

In the period from 1995 to 2019, the median yield effects of the moderate and severe drought WIs are at a similar level (−0.4 dt ha^−1^) across Germany, while the captured median yield effect for the extreme WI is 50% lower (−0.2 dt ha^−1^). Shifting the thresholds from moderate to severe intensity, a decrease in the observed number of days above the threshold (−52%) and a substantial increase in the effect size (+111%) are evident. However, a further increase from moderate to extreme intensity reduces the number of days above the threshold (−90%), displaying an approximately 50% lower yield loss than moderate or severe stress. In particular, Saxony-Anhalt, northwestern Bavaria, northeastern Baden-Wuerttemberg, almost all municipalities in Brandenburg, and parts of Mecklenburg-Western-Pomerania show the highest average yield losses (-2 to -5 dt ha^−1^). The few municipalities with positive yield effects display yield gains in the range of 2−4 dt ha^−1^ for the moderate WI, 0.01−2 dt ha^−1^ for the severe WI and 0 dt ha^−1^ for the extreme WI.

#### Heat effects during the reproductive phase (BBCH 51–75)

**Fig 7:**
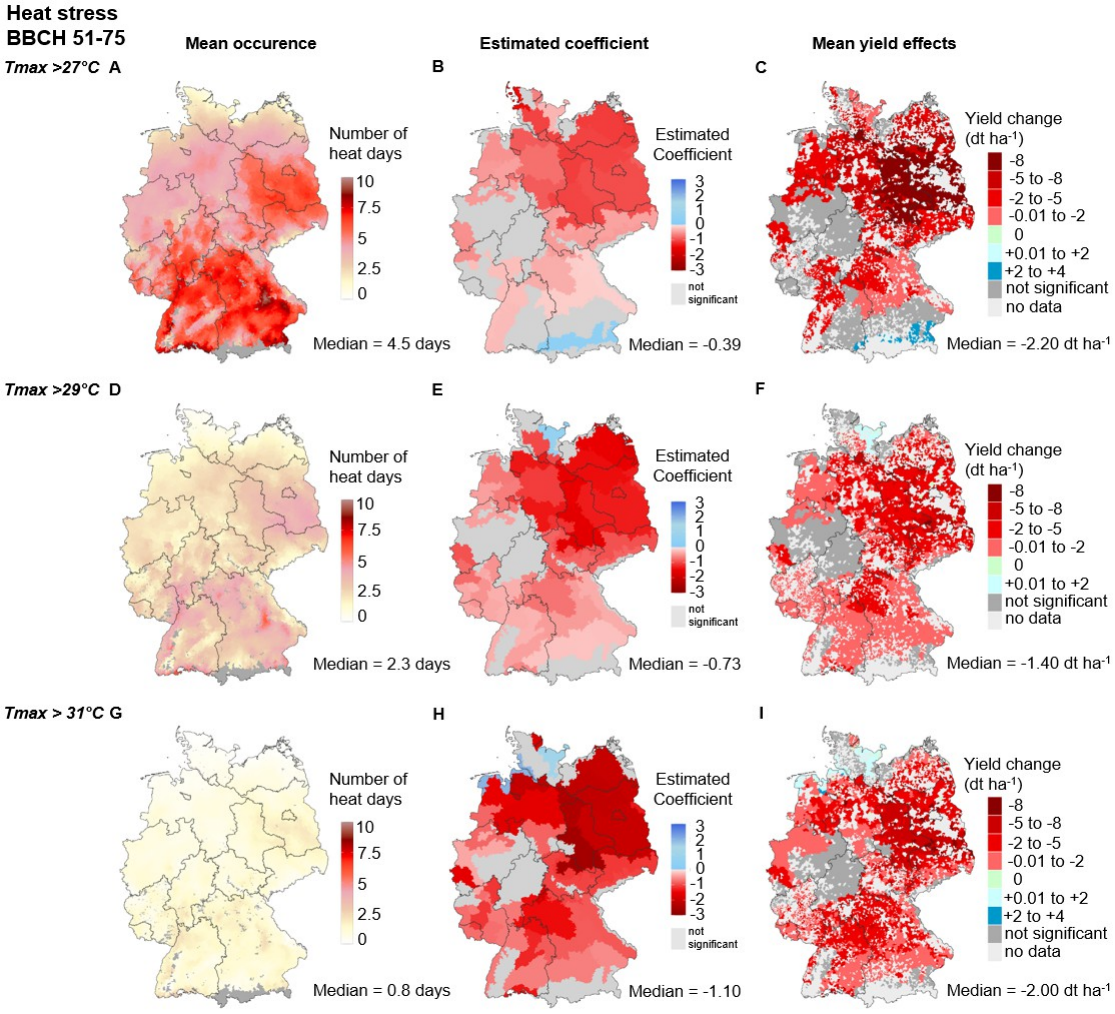
Heat stress during the reproductive phase (RP, BBCH 51–75) for moderate (*T*_*max*_ > 27°C), severe (*T*_*max*_ > 29°C) and extreme (*T*_*max*_ > 31°C) intensities. **Mean occurrence (A, D, G)** describes the average number of days above the respective thresholds between 1995 and 2019 at the municipality level. **Estimated coefficients (B, E, H)** describe the WI x SCR regression coefficients of Eq 1 for each SCR. Nonsignificant values are given in dark gray. Significant values are given in red (negative effect) or blue (positive effect). The regression coefficients and p values are displayed in Tables G-I in S1 File. **Mean yield effects (C, F, I)** describe the average yield change in dt ha^−1^ per municipality between 1995 and 2019. The median values below each map refer to the median of all municipalities’ SCRs with significant effects of the respective index. The maps were reprinted from [53] under a CC BY license, with permission from [GeoBasis-DE/ BKG], original copyright [2023].

Germany’s mean annual number of heat days during the reproductive phase ranged from 0 to 9.7 days between 1995 and 2019. The Germany-wide median decreases with increasing heat stress intensity (i.e., increasing daily maximum temperature, *T*_*max*_) from 4.5 days at *T*_*max*_ > 27°C (i.e. moderate WI) to 2.3 days at *T*_*max*_ > 29°C (i.e., severe WI), and down to 0.8 days at *T*_*max*_ > 31°C (i.e., extreme WI). During the reproductive phase, most heat days occur in eastern and southern Germany, and the fewest heat days occur along the northern coastline in Schleswig-Holstein and Mecklenburg-Western Pomerania (Fig 7A, 7C and 7G).

The median effect sizes increase with increasing intensity from −0.39 dt ha^−1^ day above threshold^−1^ at *T*_*max*_ > 27°C to −0.73 dt ha^−1^ day above threshold^−1^ at *T*_*max*_ > 29°C and up to −1.1 dt ha^−1^ day above threshold^−1^ at *T*_*max*_ > 31°C. At all heat intensities, the negative effect size is highest in the northeast and east and is lowest in the southern Germany. With increasing intensity, the regions with significant negative effect sizes increase in the south and west and decrease in the north. Consequently, the moderate WI reveals regions with nonsignificant or positive effect sizes in the south and west. In contrast, the severe and extreme WIs show positive and nonsignificant effect sizes in the north along the coastline (Fig 7B, 7E and 7H).

The median yield effects are strongest for the moderate WI (−2.2 dt ha^−1^), followed by the extreme WI (−2.0 dt ha^−1^), and are lowest for the severe WI (−1.4 dt ha^−1^). In that regard, a shift from moderate to severe intensities leads to a subtle drop in the observed number of days above the threshold (−48%) and an increase in the effect size (+87%), whereas a shift from moderate to extreme intensities leads to a substantial drop in days above the threshold (−82%) and an increase in the effect size (+182%). For all intensities, the largest yield losses appear in almost all parts of Saxony-Anhalt and Brandenburg (Fig 7C, 7F and 7I). In these regions average yield losses are greater than −8 dt ha^−1^ for moderate intensities, between -5 and -8 dt ha^−1^ for extreme intensities and between −2 and −5 dt ha^−1^ for severe intensities. The second highest yield losses are observed area-wide in Mecklenburg-Western-Pomerania (except on the coasts), in the northeastern and southern parts of Lower Saxony, in Saxony, and in northwestern Bavaria (−2 to −5 dt ha^−1^) for the moderate WI. In contrast to the north, large parts of the south (i.e., Bavaria and Baden-Wuerttemberg) show just significantly negative yield effects with severe (−0.1 to −2 dt ha^−1^) and extreme (−2 to −5 dt ha^−1^) intensities.

## Discussion

This study investigates the effects of the timing and intensity of heat and drought stress on wheat yields in Germany using WIs. For this purpose, we first study the VR of all 18 WIs to determine differences in explanatory power. Second, we analyze the region-specific effect sizes of the various WIs and identify local yield effects at the municipality level.

### Differences in explanatory power due to timing and intensity

During the reproductive phase, heat WIs generally help better explain wheat yields than drought WIs. However, this pattern is reversed during the stem elongation and booting phase, which is consistent with the findings of previous studies [20, 31, 52]. For heat WIs during the reproductive phase, a moderate stress intensity helps to explain heat-induced yield changes better than severe and extreme stress intensities. In contrast, several studies report especially strong yield effects caused by short-term, extremely high temperature stress [22, 60–64]. Nevertheless, the findings of Ben-Ari et al. [27] and Bucheli et al. [16] confirm that moderate but long-lasting and spatially uniform heat stress helps explain wheat yields in France and eastern Germany better than short-lived regional extremes.

Furthermore, during the stem elongation and booting phase, drought WIs have the highest explanatory power at moderate intensities. However, during the reproductive phase, severe and extreme drought have the highest explanatory power, which is in line with te results of previous studies [31, 65–67]. Hence, we confirm the findings from Le Gouis et al. [7] and Makinen et al. [66], which emphasize early-season droughts as particularly harmful in central Europe, where the intensity is already moderate. We also confirm the findings of Sarto et al. [68] and Schmitt et al. [14] who reported anthesis and grain filling as the most sensitive phases to heat and drought, as stress intensities increase during these phases.

Apparent differences between heat and drought stress are visible when comparing the effects of stress timing, i.e., during the generative vs. reproductive phase. The late growth phase from milk ripening to hard dough, which is excluded from the reproductive phase but included in the generative phase, is of limited relevance for yield formation. Drought stress can contribute to the ripening process in this late phenological phase and hence has no adverse yield effects. Thus, the explanatory power of the stress WI is higher during the reproductive phase than during the generative phase. Also, for the heat WIs during the generative phase, the explanatory power is two to three times higher when we restrict the observed period to the reproductive phase. Various studies confirm these findings and explicitly find a yield effect only during the reproductive phase [17, 20, 52]. Hadasch et al. [32] even found positive yield effects due to drought toward the end of the growth period for wheat in Germany.

The explanatory power of all tested WIs is small, with a maximum VR of -2.1%. In comparison, in Bönecke et al. [31], similar WIs displayed a VR of up to −25%. The difference from our study is that Bönecke et al. [31] analyze experimental data from 43 German experimental sites obtained between 1953 and 2006. Compared to our on-farm data at the municipality level, these experimental data can better reduce external effects that influence variance for two reasons: First, the point-based yield data can be linked with the respective weather and soil data with higher local resolution. Second, experimental data are obtained under rather constant and standardized management conditions. Hence, experimental data can better isolate the individual effect of WIs than our on-farm yield data from more than 10,000 practical farms, which are influenced by unknown factors related to local agronomic practices [26, 33].

### Regional yield impacts of timing and intensity

At the national level, our results reveal higher adverse yield effects due to heat than due to drought. This is confirmed by the findings of Lüttger & Feike, Trnka et al. and Zampieri et al. [13, 23, 69], who underline that heat plays a more substantial role than drought in the late growing period in Germany and Central Europe. Additionally, Semenov & Shewry [70] reported a higher risk due to heat than drought during flowering in northern Europe, as wheat matures earlier with climate change, avoiding extreme drought stress. In contrast, Schmitt et al. [14] described extreme drought as the main driver of yield losses in Germany during the reproductive phase, whereas the effect of extreme heat was not significant in their analysis. However, Schmitt et al. [14] considered only very extreme heat stress events, while we investigated moderate, severe and extreme WIs for each SCRs individually. Our approach reveals great regional differences regarding heat- and drought-related yield effects.

In that regard, the heat and drought stress-induced yield effects in our study are a function of the number of days above the threshold and the statistical effect size (i.e., estimated coefficient) of the SCR. The analysis reveals that the municipalities in Brandenburg, Saxony-Anhalt, and in northwestern Bavaria consistently show the most days above the threshold and the highest effect size per day above the threshold. Consequently, these regions display the highest yield losses with heat and drought stress off all intensities and timings, which is also in line with the findings of other studies [13, 14]. Our analysis reveals that a higher stress intensity generally accompanies (1) fewer days above the threshold and (2) a larger effect size per day above the threshold. However, there are considerable variations in the response of these two parameters. Hence, an increase in intensity leads to regionally very distinct yield effects. For example, for heat in the reproductive phase, we see almost twice as many days above the threshold in the south compared with the north at all intensities. However, we still observe much stronger adverse yield effects in the north and the east than in the south. Several studies also confirm regional variations in heat-induced yield effects. In that regard, Zampieri et al. [23] found varying yield effects for similar heat intensities at national and global levels. Additionally, Dreccer et al. [28] show that low temperatures reduce yields in western Australia, while the opposite trend dominates in norther and southern Australia.

For drought in the reproductive phase, we find relatively linear relationships between the number of days above the threshold, effect size, and yield loss. As a result, a few days above the threshold, such as in the south and west, is associated with the smallest effect size and yield losses, while in the north and east, a large number of days above the threshold accompanies large effect sizes and the highest yield losses. However, during the stem elongation and booting phase, we can see considerable regional differences in the magnitude of the effect size and yield effect. Thus, we observe a few days above the threshold and a large effect size in the north but not in the south. Lüttger & Feike [13] also revealed spatial differences in drought-induced yield effects and showed that drought stress increases yield variability in northeastern and eastern Germany. In contrast, southern Germany is consistently spared from yield-altering drought stress. Additionally, Ben-Ari et al. [27] illustrate how identical heat and drought WIs show opposite effects in France compared to Spain. They highlight that heat and drought effects differ significantly at global, national, and subnational levels.

There are several explanations regarding the regionally differing yield effects due to similar WI occurrences. First, there are reasons for natural region-specific differences in resistance to heat and drought stress that are associated with soil properties. While the simulated soil moisture data consider soil texture for deriving current plant available water, Mueller et al. and Trnka et al. [71, 72] highlight that during drought, light soils and the associated low soil water storage capacity exacerbate water stress-induced yield variations, while heavy soils may better buffer against drought stress. Furthermore, regarding heat effects, soil temperature is decisive for the yield formation of the plant [73]. Soil temperature alters the rate of organic matter decomposition and mineralization of different organic materials and soil water content, conductivity, and availability to plants [73, 74]. Soil temperature, in turn, is influenced by a wide variety of local parameters such as soil color, vegetative cover, soil mulch, the slope of the land surface, organic matter content, evaporation, solar radiation, and their interactions, and can therefore vary considerably by region [25, 74].

For the interpretation of our results, additional aspects may be considered. For example, Siebert et al. [25] discuss how the above described regionally differing yield effects of the same WI is challenging to explain from a crop physiological perspective. They emphasize that the effect of a specific WI may be influenced by (i.e., correlated with) other variables not considered in the regression analysis. This is highlighted by our results on the positive yield effects of moderate drought conditions in southern Germany. Here, the drought WI represents a proxy for a season that is not too wet, leading to drought-related yield increases in the south. In that regard, Schmitt et al. [14] show that heavy rainfall and water excess are yield-limiting factors in southern Germany, while excessively wet conditions hardly occur in northern Germany. Additionally, the heat WIs comprise other weather conditions such as drier conditions with higher incident radiation. This is supported by findings from Rezaei et al. [75], who found no heat-related yield changes in wheat, when drought stress was fully controlled. In addition, weather conditions during the growing season before or after the timing of the WIs may influence their yield effect. For example, Siebert et al. [25] and Taraz [64] emphasize that moderate stress can be (ultimately) compensated for, especially in early growth phases, if growth conditions are favorable over the rest of the growing season. In contrast, compensation in later growth phases around flowering is no longer possible in wheat, and the individual weather effect carries a more substantial weight. Additionally, compound weather events impacted spatial differences in our study, as dry and hot conditions often co-occur. For instance, the extreme 2003, 2010, and 2018 heatwaves accompanied strong drought events in central Europe [76]. Many studies highlight that the compound effects between heat and drought lead to more substantial yield losses than their isolated effects [23, 26, 76–81]. Hence, compound effects might occur only in some regions, whereas in other regions, only the isolated weather event effect shows an impact. Furthermore, individual farm management practices, which impact weather-yield relations, could not be considered in our analysis due to the lack of related data. Jones et al. [82] highlight the lack of precise knowledge of farm management practices influencing the effects of heat and drought stress, and Albers et al. and Bönecke et al. [31, 33] stress that individual farm management practices affect yield variation by more than 50%. In that regard, Macholdt & Honermeier [83] emphasize nitrate fertilization, crop rotation, and weather extremes as the most important factors influencing yield variations. Furthermore, several studies have identified plant breeding as having a primary influence on heat and drought stress-induced yield losses [26, 32, 66]. Breeding progress alters the timing of crop developmental phases [84], resource use efficiency, including water use efficiency [85], and resistance levels against biotic [9] and abiotic stress [72]. However, Albers et al. [33] highlight that detailed information on inputs would rarely alter the results in a qualitative manner, but most likely quantitatively. Finally [86] stress that interpolated weather data carry the risk of spatial autocorrelation, as errors might propagate from one grid cell to the next, resulting in significantly larger standard errors, which might affect the WI’s effect sizes. Thus, Möller et al. [29] suggest the considering local accuracy metrics, which enable a spatiotemporal quantification of interpolation errors.

## Conclusion

Building on a vast on-farm yield data set, we analyzed the effects of heat and drought on wheat yields in Germany. We specifically analyzed i) differences in explanatory power between the timing and intensity of heat and drought WIs and ii) regional differences in heat- and drought-related yield effects of winter wheat. In that regard, our mixed linear model analysis reveals the highest explanatory power for moderate heat WIs during the reproductive phase and for moderate drought WIs during the stem elongation and booting phase. Heat stress shows only limited relevance during the stem elongation and booting phase. Moreover, we find higher explanatory power when the yield-sensitive periods are defined more precisely (i.e., reproductive phase vs. generative phase). In addition, we find large regional differences in heat and drought stress-related yield effects in winter wheat. Across all WIs, we identify the strongest heat- and drought-related yield losses in the northeast and east. However, similar occurrences of heat and drought stress intensities caused much lower yield losses in other regions. Potential reasons for this finding include region- or farm-specific impacts such as genetic (G) differences (e.g., different crop varieties), environmental (E) influences (e.g., soil type) and differences in crop management (M) (e.g., sowing date). Therefore, further studies should specifically analyze G x E x M effects and respective regional interactions when analyzing crop–climate relations.

## Supporting information

S1 File (DOCX)

S1 Data (PDF)

## References

[1] Biel W, Kazimierska K, Bashutska U. Nutritional value of wheat, triticale, barley and oat grains. Acta Scientiarum Polonorum Zootechnica. 2020;19(2):19–28. doi: 10.21005/asp.2020.19.2.03

[2] Food and Agriculture Organization of the United Nations (FAO). FAOSTAT Database: Crops and livestock products; 2022. Available from: https://www.fao.org/faostat/en/#data/QCL.

[3] Shiferaw B, Smale M, Braun HJ, Duveiller E, Reynolds M, Muricho G. Crops that feed the world 10. Past successes and future challenges to the role played by wheat in global food security. Food Security. 2013;5(3):291–317. doi: 10.1007/s12571-013-0263-y

[4] Bennetzen EH, Smith P, Porter JR. Decoupling of greenhouse gas emissions from global agricultural production: 1970-2050. Global change biology. 2016;22(2):763–781. doi: 10.1111/gcb.13120 26451699

[5] Organisation for Economic Co-operation and Development (OECD) and Food and Agriculture Organization of the United Nations (FAO). OECD-FAO Agricultural Outlook 2021-2030; 2021. Available from: https://www.oecd-ilibrary.org/content/publication/19428846-en.

[6] Riedesel L, Laidig F, Hadasch S, Rentel D, Hackauf B, Piepho HP, et al. Breeding progress reduces carbon footprints of wheat and rye. Journal of Cleaner Production. 2022;377:134326. doi: 10.1016/j.jclepro.2022.134326

[7] Le Gouis J, Oury FX, Charmet G. How changes in climate and agricultural practices influenced wheat production in Western Europe. Journal of Cereal Science. 2020;93:102960. doi: 10.1016/j.jcs.2020.102960

[8] Brisson N, Gate P, Gouache D, Charmet G, Oury FX, Huard F. Why are wheat yields stagnating in Europe? A comprehensive data analysis for France. Field Crops Research. 2010;119(1):201–212. doi: 10.1016/j.fcr.2010.07.012

[9] Laidig F, Feike T, Klocke B, Macholdt J, Miedaner T, Rentel D, et al. Long-term breeding progress of yield, yield-related, and disease resistance traits in five cereal crops of German variety trials. TAG Theoretical and Applied Genetics Theoretische Und Angewandte Genetik. 2021;134(12):3805–3827. doi: 10.1007/s00122-021-03929-5 34652455 PMC8580907

[10] Zetzsche H, Friedt W, Ordon F. Breeding progress for pathogen resistance is a second major driver for yield increase in German winter wheat at contrasting N levels. Scientific Reports. 2020;10(1):20374. doi: 10.1038/s41598-020-77200-0 33230232 PMC7683597

[11] Agnolucci P, de Lipsis V. Long-run trend in agricultural yield and climatic factors in Europe. Climatic Change. 2020;159(3):385–405. doi: 10.1007/s10584-019-02622-3

[12] Gornott C, Wechsung F. Statistical regression models for assessing climate impacts on crop yields: A validation study for winter wheat and silage maize in Germany. Agricultural and Forest Meteorology. 2016;217:89–100. doi: 10.1016/j.agrformet.2015.10.005

[13] Lüttger AB, Feike T. Development of heat and drought related extreme weather events and their effect on winter wheat yields in Germany. Theoretical and Applied Climatology. 2018;132(1-2):15–29. doi: 10.1007/s00704-017-2076-y

[14] Schmitt J, Offermann F, Söder M, Frühauf C, Finger R. Extreme weather events cause significant crop yield losses at the farm level in German agriculture. Food Policy. 2022;112:102359. doi: 10.1016/j.foodpol.2022.102359

[15] Vroege W, Bucheli J, Dalhaus T, Hirschi M, Finger R. Insuring crops from space: the potential of satellite-retrieved soil moisture to reduce farmers’ drought risk exposure. European Review of Agricultural Economics. 2021;48(2):266–314. doi: 10.1093/erae/jbab010

[16] Bucheli J, Dalhaus T, Finger R. Temperature effects on crop yields in heat index insurance. Food Policy. 2022;107:102214. doi: 10.1016/j.foodpol.2021.102214

[17] Farooq M, Bramley H, Palta JA, Siddique KHM. Heat Stress in Wheat during Reproductive and Grain-Filling Phases. Critical Reviews in Plant Sciences. 2011;30(6):491–507. doi: 10.1080/07352689.2011.615687

[18] Farooq M, Hussain M, Siddique KHM. Drought Stress in Wheat during Flowering and Grain-filling Periods. Critical Reviews in Plant Sciences. 2014;33(4):331–349. doi: 10.1080/07352689.2014.875291

[19] Hlavinka P, Trnka M, Semerádová D, Dubrovský M, Žalud Z, Možný M. Effect of drought on yield variability of key crops in Czech Republic. Agricultural and Forest Meteorology. 2009;149(3-4):431–442. doi: 10.1016/j.agrformet.2008.09.004

[20] Hlaváčová M, Klem K, Rapantová B, Novotná K, Urban O, Hlavinka P, et al. Interactive effects of high temperature and drought stress during stem elongation, anthesis and early grain filling on the yield formation and photosynthesis of winter wheat. Field Crops Research. 2018;221:182–195. doi: 10.1016/j.fcr.2018.02.022

[21] Lobell DB, Hammer GL, Chenu K, Zheng B, McLean G, Chapman SC. The shifting influence of drought and heat stress for crops in northeast Australia. Global change biology. 2015;21(11):4115–4127. doi: 10.1111/gcb.13022 26152643

[22] Luo Q. Temperature thresholds and crop production: a review. Climatic Change. 2011;109(3-4):583–598. doi: 10.1007/s10584-011-0028-6

[23] Zampieri M, Ceglar A, Dentener F, Toreti A. Wheat yield loss attributable to heat waves, drought and water excess at the global, national and subnational scales. Environmental Research Letters. 2017;12(6). doi: 10.1088/1748-9326/aa723b

[24] Simpson NP, Mach KJ, Constable A, Hess J, Hogarth R, Howden M, et al. A framework for complex climate change risk assessment. One Earth. 2021;4(4):489–501. doi: 10.1016/j.oneear.2021.03.005

[25] Siebert S, Webber H, Rezaei EE. Weather impacts on crop yields—searching for simple answers to a complex problem. Environmental Research Letters. 2017;12(8):081001. doi: 10.1088/1748-9326/aa7f15

[26] Zampieri M, Ceglar A, Dentener F, Toreti A. Understanding and reproducing regional diversity of climate impacts on wheat yields: current approaches, challenges and data driven limitations. 1748-9326. 2018;13(2):021001.

[27] Ben-Ari T, Adrian J, Klein T, Calanca P, van der Velde M, Makowski D. Identifying indicators for extreme wheat and maize yield losses. Agricultural and Forest Meteorology. 2016;220:130–140. doi: 10.1016/j.agrformet.2016.01.009

[28] Dreccer MF, Fainges J, Whish J, Ogbonnaya FC, Sadras VO. Comparison of sensitive stages of wheat, barley, canola, chickpea and field pea to temperature and water stress across Australia. Agricultural and Forest Meteorology. 2018;248:275–294. doi: 10.1016/j.agrformet.2017.10.006

[29] Möller M, Doms J, Gerstmann H, Feike T. A framework for standardized calculation of weather indices in Germany. Theoretical and Applied Climatology. 2019;136(1-2):377–390. doi: 10.1007/s00704-018-2473-x

[30] Vogel E, Donat MG, Alexander LV, Meinshausen M, Ray DK, Karoly D, et al. The effects of climate extremes on global agricultural yields. 1748-9326. 2019;14(5):054010.

[31] Bönecke E, Breitsameter L, Brüggemann N, Chen TW, Feike T, Kage H, et al. Decoupling of impact factors reveals the response of German winter wheat yields to climatic changes. Global change biology. 2020;26(6):3601–3626. doi: 10.1111/gcb.15073 32154969

[32] Hadasch S, Laidig F, Macholdt J, Bönecke E, Piepho HP. Trends in mean performance and stability of winter wheat and winter rye yields in a long-term series of variety trials. Field Crops Research. 2020;252:107792. doi: 10.1016/j.fcr.2020.107792

[33] Albers H, Gornott C, Hüttel S. How do inputs and weather drive wheat yield volatility? The example of Germany. Food Policy. 2017;70:50–61. doi: 10.1016/j.foodpol.2017.05.001

[34] Conradt S, Finger R, Spörri M. Flexible weather index-based insurance design. Climate Risk Management. 2015;10:106–117. doi: 10.1016/j.crm.2015.06.003

[35] Kapphan I, Calanca P, Holzkaemper A. Climate Change, Weather Insurance Design and Hedging Effectiveness. The Geneva Papers on Risk and Insurance—Issues and Practice. 2012;37(2):286–317. doi: 10.1057/gpp.2012.8

[36] Dalhaus T, Musshoff O, Finger R. Phenology Information Contributes to Reduce Temporal Basis Risk in Agricultural Weather Index Insurance. Scientific reports. 2018;8(1):46. doi: 10.1038/s41598-017-18656-5 29311587 PMC5758701

[37] Beillouin D, Schauberger B, Bastos A, Ciais P, Makowski D. Impact of extreme weather conditions on European crop production in 2018. Philosophical transactions of the Royal Society of London Series B, Biological sciences. 2020;375(1810):20190510. doi: 10.1098/rstb.2019.0510 32892735 PMC7485097

[38] Tricker PJ, ElHabti A, Schmidt J, Fleury D. The physiological and genetic basis of combined drought and heat tolerance in wheat. Journal of Experimental Botany. 2018;69(13):3195–3210. doi: 10.1093/jxb/ery081 29562265

[39] Ray DK, Gerber JS, MacDonald GK, West PC. Climate variation explains a third of global crop yield variability. Nature communications. 2015;6:5989. doi: 10.1038/ncomms6989 25609225 PMC4354156

[40] Ben-Ari T, Boé J, Ciais P, Lecerf R, van der Velde M, Makowski D. Causes and implications of the unforeseen 2016 extreme yield loss in the breadbasket of France. Nature Communications. 2018;9(1):1627. doi: 10.1038/s41467-018-04087-x 29691405 PMC5915531

[41] Federal Ministry of Food and Agriculture (BMEL). Buchführung der Testbetriebe: Grundlagen zur BMEL—Testbetriebsbuchführung; 2018. Available from: https://www.bmel-statistik.de/fileadmin/daten/BFB-0114001-2018.pdf.

[42] German Weather Service (DWD). Historische tägliche Stationsbeobachtungen: (Temperatur, Druck, Niederschlag, Sonnenscheindauer, etc.) fürDeutschland; 2021. Available from: cdc.dwd.de/portal.

[43] German Weather Service (DWD). Dokumentation AMBAV 2.0; 2021. Available from: https://www.dwd.de/DE/fachnutzer/landwirtschaft/dokumentationen/allgemein/ambav-20_doku.html?nn=732680.

[44] Kaspar F, Zimmermann K, Polte-Rudolf C. An overview of the phenological observation network and the phenological database of Germany’s national meteorological service (Deutscher Wetterdienst). In: Advances in Science and Research. Copernicus GmbH; 2015. p. 93–99. Available from: https://asr.copernicus.org/articles/11/93/2014/.

[45] Gerstmann H, Doktor D, Gläßer C, Möller M. PHASE: A geostatistical model for the Kriging-based spatial prediction of crop phenology using public phenological and climatological observations. Computers and Electronics in Agriculture. 2016;127:726–738. doi: 10.1016/j.compag.2016.07.032

[46] Siebert S, Ewert F. Spatio-temporal patterns of phenological development in Germany in relation to temperature and day length. Agricultural and Forest Meteorology. 2012;152:44–57. doi: 10.1016/j.agrformet.2011.08.007

[47] Möller M, Boutarfa L, Strassemeyer J. PhenoWin—An R Shiny application for visualization and extraction of phenological windows in Germany. Computers and Electronics in Agriculture. 2020;175:105534. doi: 10.1016/j.compag.2020.105534

[48] Möller M, Gerstmann H, Gao F, Dahms TC, Förster M. Coupling of phenological information and simulated vegetation index time series: Limitations and potentials for the assessment and monitoring of soil erosion risk. CATENA. 2017;150:192–205. doi: 10.1016/j.catena.2016.11.016

[49] Federal Institute for Geosciences and Natural Resources (BGR). Nutzungsdifferenzierte Bodenübersichtskarte der Bundesrepublik Deutschland: BÜK 1000 N; 2007. Available from: https://www.bgr.bund.de/DE/Themen/Boden/Informationsgrundlagen/Bodenkundliche_Karten_Datenbanken/BUEK1000/buek1000_node.html.

[50] Federal Agency for Cartography and Geodesy (BKG). Dokumentation: Digitaes Basis-Landschaftsmodell: ATKIS-Objektartenkatalog Basis-DLM; 2018.

[51] Dalhaus T, Finger R. Can Gridded Precipitation Data and Phenological Observations Reduce Basis Risk of Weather Index–Based Insurance? Weather, Climate, and Society. 2016;8(4):409–419. doi: 10.1175/WCAS-D-16-0020.1

[52] Wollenweber B, Porter JR, Schellberg J. Lack of Interaction between Extreme High-Temperature Events at Vegetative and Reproductive Growth Stages in Wheat. Journal of Agronomy and Crop Science. 2003;189(3):142–150. doi: 10.1046/j.1439-037X.2003.00025.x

[53] Federal Agency for Cartography and Geodesy (BKG). NUTS-Gebiete: NUTS5000; 2023. Available from: https://gdz.bkg.bund.de/index.php/default/open-data/nuts-gebiete-1-5-000-000-stand-31-12-nuts5000-31-12.html.

[54] Steduto P, Hsiao TC, Raes D, Fereres E. AquaCrop—The FAO Crop Model to Simulate Yield Response to Water: I. Concepts and Underlying Principles. Agronomy Journal. 2009;101(3):426–437. doi: 10.2134/agronj2008.0139s

[55] Roßberg D, Michel V, Graf R, Neukampf R. Definition von Boden-Klima-Räumen für die Bundesrepublik Deutschland; 2007. Available from: https://www.openagrar.de/receive/openagrar_mods_00056830.

[56] Piepho HP. A coefficient of determination (R2) for generalized linear mixed models. Biometrical journal Biometrische Zeitschrift. 2019;61(4):860–872. 30957911 10.1002/bimj.201800270

[57] Akaike Htrotugu. Maximum likelihood identification of Gaussian autoregressive moving average models. Biometrika. 1973;60(2):255–265. doi: 10.1093/biomet/60.2.255

[58] Faraway JJ. Extending the linear model with R: generalized linear, mixed effects and nonparametric regression models. Chapman and Hall/CRC; 2016.

[59] Sheather S. A Modern Approach to Regression with R. SpringerLink Bücher. New York, NY: Springer New York; 2009.

[60] Asseng S, Foster IAN, Turner NC. The impact of temperature variability on wheat yields. Global change biology. 2011;17(2):997–1012. doi: 10.1111/j.1365-2486.2010.02262.x

[61] Dias AS, Lidon FC. Evaluation of Grain Filling Rate and Duration in Bread and Durum Wheat, under Heat Stress after Anthesis. Journal of Agronomy and Crop Science. 2009;195(2):137–147. doi: 10.1111/j.1439-037X.2008.00347.x

[62] Ferris R. Effect of High Temperature Stress at Anthesis on Grain Yield and Biomass of Field-grown Crops of Wheat. Annals of Botany. 1998;82(5):631–639. doi: 10.1006/anbo.1998.0740

[63] Tashiro T, Wardlaw IF. The Response to High Temperature Shock and Humidity Changes Prior to and During the Early Stages of Grain Development in Wheat. Functional Plant Biology. 1990;17(5):551. doi: 10.1071/PP9900551

[64] Taraz V. Can farmers adapt to higher temperatures? Evidence from India. World Development. 2018;112:205–219. doi: 10.1016/j.worlddev.2018.08.006

[65] Kato Y, Kamoshita A, Yamagishi J. Preflowering Abortion Reduces Spikelet Number in Upland Rice (Oryza sativa L.) under Water Stress. Crop Science. 2008;48(6):2389–2395. doi: 10.2135/cropsci2007.11.0627

[66] Mäkinen H, Kaseva J, Trnka M, Balek J, Kersebaum KC, Nendel C, et al. Sensitivity of European wheat to extreme weather. Field Crops Research. 2018;222:209–217. doi: 10.1016/j.fcr.2017.11.008

[67] Varga B, Vida G, Varga-László E, Bencze S, Veisz O. Effect of Simulating Drought in Various Phenophases on the Water Use Efficiency of Winter Wheat. Journal of Agronomy and Crop Science. 2015;201(1):1–9. doi: 10.1111/jac.12087

[68] Sarto MVM, Sarto JRW, Rampim L, Rosset JS, Bassegio D, da Costa PF, et al. Wheat phenology and yield under drought: a review. Australian Journal of Crop Science. 2017;11(8):941–946. doi: 10.21475/ajcs.17.11.08.pne351

[69] Trnka M, Brázdil R, Olesen JE, Eitzinger J, Zahradníček P, Kocmánková E, et al. Could the changes in regional crop yields be a pointer of climatic change? Agricultural and Forest Meteorology. 2012;166-167:62–71. doi: 10.1016/j.agrformet.2012.05.020

[70] Semenov MA, Shewry PR. Modelling predicts that heat stress, not drought, will increase vulnerability of wheat in Europe. Scientific Reports. 2011;1(1):66. doi: 10.1038/srep00066 22355585 PMC3216553

[71] Mueller L, Schindler U, Shepherd T, Ball B, Smolentseva E, Pachikin K, et al. In: Mueller L, Saparov A, Lischeid G, editors. The Muencheberg Soil Quality Rating for assessing the quality of global farmland. Springer International; 2014. p. 235–248.

[72] Trnka M, Rötter RP, Ruiz-Ramos M, Kersebaum KC, Olesen JE, Žalud Z, et al. Adverse weather conditions for European wheat production will become more frequent with climate change. Nature Climate Change. 2014;4(7):637–643. doi: 10.1038/nclimate2242

[73] Mondal S, Ghosal S, Barua R. Impact of elevated soil and air temperature on plants growth, yield and physiological interaction: a critical review. Scientia Agriculturae. 2016;14(3).

[74] Onwuka BM. Effects of soil temperature on some soil properties and plant growth. Scholarly Journal of Agricultural Science. 2016;6(3):89–93.

[75] Rezaei EE, Siebert S, Manderscheid R, Müller J, Mahrookashani A, Ehrenpfordt B, et al. Quantifying the response of wheat yields to heat stress: The role of the experimental setup. Field Crops Research. 2018;217:93–103. doi: 10.1016/j.fcr.2017.12.015

[76] Ribeiro AFS, Russo A, Gouveia CM, Páscoa P, Zscheischler J. Risk of crop failure due to compound dry and hot extremes estimated with nested copulas. Biogeosciences. 2020;17(19):4815–4830. doi: 10.5194/bg-17-4815-2020

[77] Lesk C, Coffel E, Winter J, Ray D, Zscheischler J, Seneviratne SI, et al. Stronger temperature–moisture couplings exacerbate the impact of climate warming on global crop yields. Nature Food. 2021;2(9):683–691. doi: 10.1038/s43016-021-00341-6 37117467

[78] Lobell DB, Bänziger M, Magorokosho C, Vivek B. Nonlinear heat effects on African maize as evidenced by historical yield trials. Nature Climate Change. 2011;1(1):42–45. doi: 10.1038/nclimate1043

[79] Lobell DB, Schlenker W, Costa-Roberts J. Climate trends and global crop production since 1980. Science (New York, NY). 2011;333(6042):616–620. doi: 10.1126/science.1204531 21551030

[80] Lobell DB, Sibley A, Ivan Ortiz-Monasterio J. Extreme heat effects on wheat senescence in India. Nature Climate Change. 2012;2(3):186–189. doi: 10.1038/nclimate1356

[81] Zscheischler J, Westra S, van den Hurk BJJM, Seneviratne SI, Ward PJ, Pitman A, et al. Future climate risk from compound events. Nature Climate Change. 2018;8(6):469–477. doi: 10.1038/s41558-018-0156-3

[82] Jones JW, Antle JM, Basso B, Boote KJ, Conant RT, Foster I, et al. Toward a new generation of agricultural system data, models, and knowledge products: State of agricultural systems science. Agricultural Systems. 2017;155:269–288. doi: 10.1016/j.agsy.2016.09.021 28701818 PMC5485672

[83] Macholdt J, Honermeier B. Stability analysis for grain yield of winter wheat in a long-term field experiment. Archives of Agronomy and Soil Science. 2019;65(5):686–699. doi: 10.1080/03650340.2018.1520979

[84] Olesen JE, Børgesen CD, Elsgaard L, Palosuo T, Rötter RP, Skjelvåg AO, et al. Changes in time of sowing, flowering and maturity of cereals in Europe under climate change. Food additives & contaminants Part A, Chemistry, analysis, control, exposure & risk assessment. 2012;29(10):1527–1542. doi: 10.1080/19440049.2012.712060 22934894

[85] Snowdon RJ, Wittkop B, Chen TW, Stahl A. Crop adaptation to climate change as a consequence of long-term breeding. TAG Theoretical and Applied Genetics Theoretische Und Angewandte Genetik. 2021;134(6):1613–1623. doi: 10.1007/s00122-020-03729-3 33221941 PMC8205907

[86] Auffhammer M, Hsiang SM, Schlenker W, Sobel A. Using Weather Data and Climate Model Output in Economic Analyses of Climate Change. 2013;(19087).

