# Supplementary material for: Timing and intensity of heat and drought stress determine wheat yield losses in Germany
Source: PLoS One. 2023 Jul 25;18(7):e0288202. doi: 10.1371/journal.pone.0288202 (PMC10368287; doi:10.1371/journal.pone.0288202)
Supplement: S1 File — (DOCX) [file pone.0288202.s001.docx]

Supporting information

S1 Table. Table A - SCR. Definition of the SCR Number with English translation and original German designations. File: https://doi.org/10.5281/zenodo.8076295

S1 Table. Table B - VR WI. Variance reduction (VR) of selected heat and drought WIs. File: https://doi.org/10.5281/zenodo.8076295

S1 Table. Table C - VR FE. Variance reduction (VR) and variance inflation factor (VIF) of covariables that were tested to be included/excluded into/from the final model as fixed effects (FE). File: https://doi.org/10.5281/zenodo.8076295

S1 Table. Table D - H27 GP. Model output of Eq. (1) with the covariate H27 GP as the WI term (wjl) under evaluation. File: https://doi.org/10.5281/zenodo.8076295

S1 Table. Table E - H29 GP. Model output of Eq. (1) with the covariate H29 GP as the WI term (wjl) under evaluation. File: https://doi.org/10.5281/zenodo.8076295

S1 Table. Table F - H31 GP. Model output of Eq. (1) with the covariate H31 GP as the WI term (wjl) under evaluation. File: https://doi.org/10.5281/zenodo.8076295

S1 Table. Table G - H27 RP. Model output of Eq. (1) with the covariate H27 RP as the WI term (wjl) under evaluation. File: https://doi.org/10.5281/zenodo.8076295

S1 Table. Table H - H29 RP. Model output of Eq. (1) with the covariate H29 RP as the WI term (wjl) under evaluation. File: https://doi.org/10.5281/zenodo.8076295

S1 Table. Table I - H31 RP. Model output of Eq. (1) with the covariate H31 RP as the WI term (wjl) under evaluation. File: https://doi.org/10.5281/zenodo.8076295

S1 Table. Table J - D50 GP. Model output of Eq. (1) with the covariate D50 GP as the WI term (wjl) under evaluation. File: https://doi.org/10.5281/zenodo.8076295

S1 Table. Table K - D30 GP. Model output of Eq. (1) with the covariate D30 GP as the WI term (wjl) under evaluation. File: https://doi.org/10.5281/zenodo.8076295 July 16, 2023 15/23

S1 Table. Table L - D10 GP. Model output of Eq. (1) with the covariate D10 GP as the WI term (wjl) under evaluation. File: https://doi.org/10.5281/zenodo.8076295

S1 Table. Table M - D50 RP. Model output of Eq. (1) with the covariate D50 RP as the WI term (wjl) under evaluation. File: https://doi.org/10.5281/zenodo.8076295

S1 Table. Table N - D30 RP. Model output of Eq. (1) with the covariate D30 RP as the WI term (wjl) under evaluation. File: https://doi.org/10.5281/zenodo.8076295

S1 Table. Table O - D10 RP. Model output of Eq. (1) with the covariate D10 RP as the WI term (wjl) under evaluation. File: https://doi.org/10.5281/zenodo.8076295

S1 Table. Table P - D50 SEB. Model output of Eq. (1) with the covariate D50 SEB as the WI term (wjl) under evaluation. File: https://doi.org/10.5281/zenodo.8076295

S1 Table. Table Q - D30 SEB. Model output of Eq. (1) with the covariate D30 SEB as the WI term (wjl) under evaluation. File: https://doi.org/10.5281/zenodo.8076295

S1 Table. Table R - D10 SEB. Model output of Eq. (1) with the covariate D10 SEB as the WI term (wjl) under evaluation. File: https://doi.org/10.5281/zenodo.8076295

S1 Table. Table S - H27 SEB. Model output of Eq. (1) with the covariate H27 SEB as the WI term (wjl) under evaluation. File: https://doi.org/10.5281/zenodo.8076295

S1 Table. Table T - H29 SEB. Model output of Eq. (1) with the covariate H29 SEB as the WI term (wjl) under evaluation. File: https://doi.org/10.5281/zenodo.8076295

S1 Table. Table U - H31 SEB. Model output of Eq. (1) with the covariate H31 SEB as the WI term (wjl) under evaluation. File: https://doi.org/10.5281/zenodo.8076295

S1 Table. Table V - Minimal Dataset. Minimal dataset replicate this study’s findings and figures. File: https://doi.org/10.5281/zenodo.8076295
